# B cell diversification in gut-associated lymphoid tissues: From birds to humans

**DOI:** 10.1084/jem.20231501

**Published:** 2023-10-12

**Authors:** Jean-Claude Weill, Sandra Weller, Claude-Agnès Reynaud

**Affiliations:** 1 Université Paris Cité, Institut national de la santé et de la recherche médicale U1151, Centre national de la recherche scientifique UMR-8253, Institut Necker Enfants Malades, Paris, France

## Abstract

Several species generate their preimmune repertoire in gut-associated lymphoid tissues (GALT), compensating a reduced germline V gene repertoire by post-rearrangement diversification mechanisms (gene conversion and/or somatic hypermutation) in these environments that act as primary lymphoid organs. We summarize here these processes for three different species (chickens, sheep, and rabbits) and further discuss the analogous process that T-independent B cell responses in humans represent: we indeed recently showed that response against bacterial polysaccharides mobilize marginal zone B cells that prediversified against gut antigens. While the initial diversification strategy differs in these two cases, i.e., repertoire formation driven by gut-derived mitotic signals vs. response against gut antigens, the common feature of these two processes is the mobilization of a B cell compartment prediversified in GALT for immune responses against distinct systemic antigens.

## Teaser

The authors discuss here B cell activation processes taking place in GALT, repertoire diversification in species like chicken, sheep, or rabbit versus responses to gut flora in humans, both allowing systemic responses against distinct antigens, notably bacterial polysaccharides in humans.

## Introduction

Jacques Monod, one of the fathers of molecular biology, used to advise his young colleagues to work on a paradox, an observation in contradiction with current knowledge. The immune response to encapsulated bacteria in humans is one such paradox. Effectively, within 1 wk of vaccination against these highly pathogenic microbes (pneumococcus, meningococcus, and *Haemophilus influenzae*), an immune response composed of protective, high-affinity, highly mutated antibodies is induced (Galson et al., 2015; Zhou et al., 2002). This is paradoxical because these bacteria are encased in a polysaccharide capsule that cannot be presented by MHC molecules and cannot, therefore, induce a cognate T–B collaboration leading to a germinal center (GC) reaction and an ongoing somatic hypermutation (SHM) process. One possible explanation put forward for the antibody response to these pathogens was the prediversification by hypermutation and, thus, preactivation of the B cells responding to these bacteria, enabling them to produce appropriate antibodies within a few days (Weller et al., 2004). However, this explanation raises another obvious question: When and where does this prediversification take place, and which B cells are involved?

## Of chickens, sheep, and rabbits

The diversity of the lymphocytes of the adaptive immune system remained a major enigma for decades until the discovery by Susumu Tonegawa that the roulette of random rearrangement involving a few hundred V, D, J genes to a unique constant region together with junctional diversification could produce an almost infinite number of different B cell receptors (BCRs), each BCR being composed of a membrane-bound antibody molecule (Tonegawa, 1983). This process was shown to occur in the bone marrow throughout life, with this ongoing differentiation ensuring a constant output of B cells with new receptor combinations. The beauty of these discoveries in mice was that they proved to be largely applicable to human B cells too. As pointed out by Melvin Cohn in his conferences (Cohn et al., 1980), this random diversity would make it possible to deal with any unknown and unanticipated antigenic structure, even “moondust.” Another clear implication of this finding is that many of these BCRs would recognize self-structures and would therefore need to be purged from the system as soon as they are produced. Evolution had thus developed a sophisticated toolbox, and the expectation was that all species with an adaptive immune system would make use of it.

The chicken immune system was intriguing in this context because generation of the B cell compartment in this species requires the bursa of Fabricius, an organ present in all birds at the end of the digestive tract, close to the cloaca, that undergoes involution in response to hormonal signaling several months after hatching. A few 10,000 cells that have undergone productive heavy and light chain (V_H_/V_L_) rearrangement migrate to the epithelium of the bursa at an early embryonic stage and proliferate within bursal follicles (10,000 follicles, each with 1–10 founder cells) before and several weeks after hatching (reviewed in Ratcliffe, 2006). At the molecular level, the unique organization of the chicken Ig locus, with a single functional V_H_/V_L_ pair and a large family of upstream pseudogenes, proved a major surprise (Reynaud et al., 1985, 1989). An ongoing gene conversion process between the pseudogenes and the single V_H_/V_L_ acceptor genes in the embryonic and postnatal bursa was found to produce a large population of diversified B cells, which migrated out of the bursa to establish the peripheral B cell compartment. Evolution has selected pseudogenes, which do not possess functional promoters or recombination signals but have framework regions homologous to the functional acceptor gene and highly diverse complementarity-determining regions (CDRs), to allow homology-mediated recombination to take place. Moreover, most V_H_ pseudogenes are fused V-D elements, allowing gene conversion to proceed in the CDR3 region (Reynaud et al., 1987, 1989). This process appears to be the equivalent in nature to the CDR grafting pioneered by Greg Winter in vitro to humanize mouse antibodies (Jones et al., 1986). A similar rearrangement involving a unique V_L_ gene was observed in most birds such as quail, mallard duck, turkey, and hawk, while the Muscovy duck appears to use two functional V_L_ genes (McCormack et al., 1989). A structure equivalent to the chicken bursa was subsequently identified in some mammalian species. Reynolds and Morris had shown that the Peyer’s patches of sheep were of two types: ileal Peyer’s patches, which behave functionally like a primary lymphoid organ, i.e., a lymphoid site in which the preimmune repertoire is generated, and jejunal Peyer’s patches, which display all the properties of a secondary lymphoid organ (and of mouse Peyer’s patches), in which immune responses take place (Reynolds and Morris, 1983). B cell progenitors (a few millions) expressing a productive V_H_/V_L_ rearrangement were shown to migrate to the ileal Peyer’s patch epithelium during fetal development and to proliferate for several months within follicles (around 100,000 in the 1-m length of lymphoid tissue running along the sheep ileum). Ig gene rearrangement occurs during a short period of fetal development, essentially in the fetal spleen and liver. The major light chain locus (λ) in sheep contains about 100 genes, only about half of which are functional, with two of these genes supplying 50% of the functional rearranged light chains. During proliferation, B cells diversify their BCRs by SHM through a process similar to that occurring during affinity maturation in T-dependent immune responses (Reynaud et al., 1991, 1995). Like for gene conversion, this diversification is triggered by the mutagenic enzyme activation-induced cytidine deaminase (AID; Schatz, 2007). It begins during fetal development and continues for several months after birth, until the ileal Peyer’s patches involute. By contrast, the jejunal Peyer’s patches remain in place throughout life. During this involution, the diversified B cells leave the gut and establish the peripheral B cell population. Experiments on germ-free sheep and sterile ileal loops isolated from the gut at fetal stages showed that gut bacterial antigens provide B cells with a stimulus for proliferation rather than a cognate BCR signal. Moreover, thymectomy on the embryo did not impair the SHM process, which proceeded normally in the absence of T cells (Reynaud et al., 1995). V gene mutations were essentially clustered in the CDRs, even in the absence of bacteria, thus questioning the mechanism behind this selection. The preferential presence of AID hotspots in the CDRs, as exemplified by the differential usage of serine codons between frameworks and CDRs, explained this surprising phenomenon. Massive negative selection must take place during this process of B cell production because only about 5% of B cells produced in situ migrate to the periphery (Reynolds, 1986). A cellular and molecular strategy involving intense proliferation under non-cognate stimulation by bacteria in the gut, i.e., in the absence of T–B interactions through MHC antigen presentation, has thus evolved in sheep, together with a specific molecular diversification mechanism targeting selected V gene sequences (Reynaud et al., 1991, 1995).

However, another chapter was added to this story when Kathrin Knight and her colleagues reported that the preimmune repertoire of rabbits is also generated in gut-associated lymphoid tissues (GALT), ileal Peyer’s patches, sacculus rotundus, and appendix. They showed that the heavy chain locus was composed of a large family of functional V_H_ genes, all of the V_H_3 family, and that the most proximal V_H_ gene was rearranged in 80% of B cells (Becker and Knight, 1990). This V_H_ gene is then diversified by gene conversion, with upstream functional or non-functional V_H_ genes as the donor. Interestingly, the heavy chain CDR3 region was also diversified by SHM in rabbits. As in previous models, rearrangement occurred during fetal development, but in rabbits, proliferation within follicles does not begin until after birth, once the gut has been colonized by bacteria. Strikingly, these gut-associated primary lymphoid organs do not undergo involution in rabbits, but instead develop into secondary lymphoid organs several months after birth once the peripheral B cell repertoire is established. As observed in sheep, the gut constituents essentially provide a mitotic stimulus rather than a cognate BCR-mediated signal (Rhee et al., 2004; Becker and Knight, 1990). No de novo production of naive B cells was observed at the adult stage in any of these models. This implies that naive B cells undergo self-renewal or are continually replenished by progenitors with stem cell properties. During secondary responses to T-dependent antigens, further diversification occurs in GCs, with a reactivation of SHM and/or gene conversion processes.

**Figure 1.**
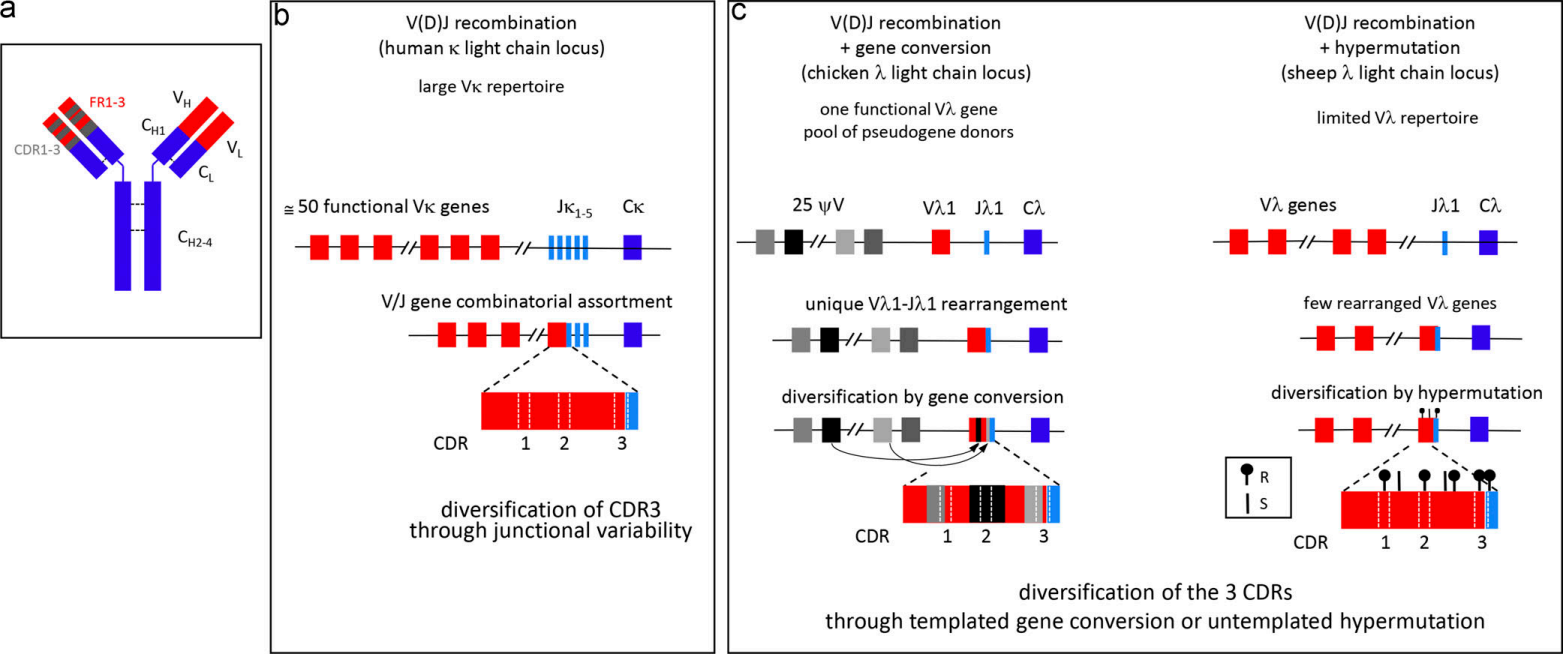
**Preimmune repertoire formation in humans versus GALT species for their major light chain isotype (κ light chain locus for humans, λ light chain locus for chickens and sheep). (a)** Schematic representation of an Ig molecule with variable (V) and constant (C) regions of the heavy (H) and light (L) chains. On the left arm of the Ig, position of framework (FR) and CDRs are represented. **(b)** Formation of the preimmune repertoire in humans, with V(D)J recombination providing Vκ and Jκ combinatorial assortment. Junctional variability has only a moderate impact on CDR3 diversification. **(c)** Formation of the preimmune repertoire by post-rearrangement diversification mechanisms, gene conversion in chickens (left), and SHM in sheep (right). In chickens, a unique Vλ1-Jλ1 rearrangement is diversified by gene conversion encompassing the complete V sequence using a pool of upstream pseudogenes (ψV) as donors. In sheep, a limited number of functional, rearranged Vλ genes are diversified by SHM, with high incidence of replacement (R) mutations in CDRs through biased codon usage and of silent (S) mutation in FRs through negative selection against amino acid changes (see text).

Among mammals, rabbit is the only species for which a role of gene conversion in formation of the B cell repertoire has been formally demonstrated. We have coined the term “GALT species” for all species in which the preimmune B cell repertoire is generated in the GALT (Weill and Reynaud, 1992; Reynaud and Weill, 1996). Both processes—gene conversion and SHM—are highly efficient at generating a preimmune repertoire, as, unlike V(D)J recombination, they affect all three CDRs (Fig. 1). The efficiency of gene conversion stems from the selective pressure imposed on pseudogenes (or donor genes) with conserved framework regions and diverse CDR sequences. Hypermutation appears to be equally efficient in the absence of antigenic selection pressure through the clustering of AID hotspots by codon selection in CDRs and BCR-dependent counterselection against deleterious mutations in framework regions.

Throughout evolution, the adaptative immune system, which arose 500 million years ago in jawed vertebrates (including cartilaginous and bony fishes, amphibians, reptiles, birds, and mammals) does not show a uniform pattern. This is particularly true for the B cell compartment, which, despite displaying in all species an almost unlimited diversity of assembled antigen receptors, uses different strategies of preimmune repertoire diversification. Some species have evolved a high number of variable genes with a large combinatorial diversity provided by V(D)J recombination (mice, humans, amphibians, cartilaginous and bony fishes), while others use very few assembled V genes further diversified by post-rearrangement processes such as gene conversion and/or SHM (birds, sheep, rabbits; Flajnik, 2002; Fig. 1). Many other mammalian species (cattle, horses, swine) most probably belong to the latter group since they possess a limited V gene repertoire with marks of prediversification, but it is not yet formally established that these events also take place in GALTs (Wang et al., 2013; Verma and Aitken, 2012; Sinclair et al., 1997; Butler, 1998).

## Of mice and men, but mostly humans

The evolution of a specific subset of B cells in the marginal zone (MZ) of the spleen with a preactivated state allowing them to respond rapidly to repetitive glycan structures in the absence of cognate T cell help has been described in detail in mice. The antibodies secreted by this response are essentially of the IgM and IgG3 isotype, and the V genes involved are in their germline configuration without SHM (Martin et al., 2001). A landmark paper from the group of Klaus Rajewsky showed that in humans, contrary to what has been observed in mice, memory B cells carrying a mutated BCR and expressing the CD27 receptor, a member of the tumor necrosis factor superfamily, on their surface, account for about 40% of the peripheral B cell population (Klein et al., 1998). The observation was novel in that half of these CD27^+^ B cells were found to harbor an IgM/IgD isotype. We further characterized this IgM/IgD B cell subset in humans. These cells were found to have similar characteristics to the mouse MZ B cell population, but with a number of clear differences. These B cells are located in the MZ region of the spleen and have an MZ B cell phenotype: IgM^high^, IgD^low^, CD21^+^, CD23^−^, CD27^+^, and CD1c^high^ (whereas mouse MZ B cells are CD1d^+^), and they account for about 15% of total B cells. Unlike their murine counterparts, these MZ-like B cells recirculate in the blood and their BCRs carry large numbers of somatic mutations. These cells are present and mutated in CD40/40L-deficient patients without GCs and switched memory B cells, but in smaller numbers and with lower overall levels of V gene mutation. They also develop and diversify in infants displaying no signs of antigen-driven clonal expansion, at a time when T-independent responses are not yet functional (Weill et al., 2009). Furthermore, in several studies, a low frequency of IgM^+^IgD^+^CD27^+^ B cells has been shown to be associated with impaired protective immunity to pneumococcal infections (Kruetzmann et al., 2003). Despite these findings, the presence of cells equivalent to the murine MZ B cell subset in humans has remained controversial. It has been argued that these cells have a phenotype similar to that of classical memory B cells and that they often share clonal relationships with switched memory B cells. Moreover, they frequently harbor a mutated *Bcl6* gene, a hallmark of the off-target mutagenic activity of AID in GCs, suggesting that they might originate from such a differentiation pathway. Based on these results, it was concluded that these cells were probably classical memory B cells involved in a T-dependent response, leaving the GC before switching to other isotypes (Tangye and Good, 2007; Seifert and Küppers, 2009; Budeus et al., 2015). We went beyond simple correlative descriptions by studying healthy volunteers vaccinated with the non-conjugated pneumococcal vaccine Pneumovax (containing 23 different pneumococcal serotypes) to determine which B cells were mobilized during the response (Weller et al., 2023). A strong plasma cell (PC) response to the 23 serotypes was observed in the blood 7 days after vaccination. Each PC clone comprised the IgM, IgG, and IgA isotypes, and all the clones had similar numbers of V_H_ gene mutations (about 20), many associated with very large clonal expansions. An analysis of all B cell subsets before vaccination demonstrated that responsive PC clones displayed predominantly clonal relationships with MZ B cells with a similar frequency of V_H_ gene mutations. These MZ B cells present before vaccination switched to other isotypes after immunization, with a trend toward IgA, and many of them had already undergone switching before immunization. 8 weeks after vaccination, no accumulation of mutations was observed in the responsive clones, in contrast to what occurs in classical T-dependent responses. There was also no detectable attrition of the B cell clones involved in the major PC expansion observed on day 7. This response to Pneumovax vaccination had a characteristic T-independent profile, with a predominance of the IgG2, IgA1, and IgA2 isotypes. Strikingly, such a T-independent profile was already observed before vaccination among clones sharing multiple isotypes.

GC responses in Peyer’s patches are largely generated by classical T–B interactions driven by gut microbial antigens. However, one cannot exclude that the antibody polarization we observed could be initiated by non-canonical T cell help, which was reported to also take place in Peyer’s patches, the constant stimulation provided by the high load of gut antigens, making it possible to circumvent cognate T–B interactions (Biram and Shulman, 2020). Several non-mutually exclusive activation processes including TLR–BCR co-engagement (Rivera et al., 2023) and bystander cytokine activation (Gregoire et al., 2022) could also occur during classical T–B responses against glycoprotein structures in the Peyer’s patch cytokinic context, thus accounting for this “T-independent-like” antibody profile. These results confirmed our initial hypothesis that the B cells responding to a T-independent vaccine were prediversified before vaccination (Weller et al., 2023). We then addressed the question as to which antigens drove this prediversification process. We re-expressed 28 antibodies recognizing different pneumococcus serotypes and showed that they were able to recognize commensal bacteria from the four major phyla (Actinobacteria, Bacteroides, Firmicutes, and Proteobacteria), each antibody recognizing a unique combination of bacteria from different phyla and genera, with different affinities. These antibodies were not polyreactive, but reversion to their germline configuration resulted in a loss of specificity against their respective pneumococcal serotypes, but, with various degrees, in maintenance of their recognition of commensal microbes. Taking into account the findings of the group of Jo Spencer that MZ B cell precursors migrate to the gut where they differentiate into MZ B cells before returning to the periphery (Tull et al., 2021), we suggested that this differentiation was probably driven by gut bacterial glycans and that, following vaccination or infection with encapsulated bacteria, B cells with the appropriate specificity were stimulated and gave rise to antibody-secreting cells. In accordance with this model, it has been shown in MyD88- and IRAK4-deficient patients, who have abnormally low levels of MZ B cells (Weller et al., 2012), that the decrease in this population of cells is correlated with impaired bacterial glycan recognition by circulating antibodies (Maglione et al., 2014).

The picture as of today in human GALT often features IgM-only memory and MZ B cells as separate entities, which both can switch to other isotypes and diversify their BCR in Peyer’s patch–GCs (Magri et al., 2017; Zhao et al., 2018). However, this issue does not seem totally settled since circulating MZ B cells and IgM-only memory B cells present overlapping repertoires (Weller et al., 2023). MZ B cells, which originate from a splenic precursor will, after their step in GALT, migrate back to the splenic MZ to respond to circulating T-independent antigens (Descatoire et al., 2014; Zhao et al., 2018; Tull et al., 2021). Along with others, we have reported that switched memory B cells derived from T-dependent responses acquire some MZ B cells markers and most probably transit in the splenic MZ (Kibler et al., 2021; Ettinger et al., 2007; Chappert et al., 2022). However, careful phenotypic analysis showed that these cells differ from MZ B cells since they do not display some of their distinctive properties such as the activation of the mTOR signaling pathway and the expression of CD148 (Gaudette et al., 2021; Cox et al., 2023; Kibler et al., 2021; Sintes et al., 2017; Chappert et al., 2022).

Many unanswered questions remain concerning human MZ B cells. Do they undergo continual repopulation by precursor cells or are they self-maintained? The group of Antonio Lanzavecchia recently reported that, in a longitudinal analysis over several years, the most expanded long-lived B cell clones in the blood of healthy individuals displayed a T-independent profile (Phad et al., 2022). It is tempting to speculate that these persistent B cell clones may at least partly correspond to the MZ B cells elicited by recurrent exposure to bacterial glycans. Nevertheless, the reappearance of MZ B cells over time following bone marrow transplantation also suggests that this population can be regenerated in certain settings (Avanzini et al., 2005). Other unanswered questions relate to the possible alternative functions of MZ B cells, such as shuttling blood T-dependent antigens to B cell follicles, as shown in mice (Cinamon et al., 2008). Could they be targeted in vaccination strategies against pathogens bearing neutralizable glycan epitopes (Williams et al., 2021)? Finally, are they involved in certain diseases, such as lupus, in which these cells are strongly reduced whereas all the other B cell subsets are present at normal levels (Wehr et al., 2004)?

In conclusion, these results show that a subset of B cells undergoing BCR diversification in the GALT has evolved in humans and are reminiscent of the observations reported by us and others for GALT species. However, there are clear differences. In particular, gut bacterial glycans appear to exert positive selection pressure on the diversification process of MZ B cells in humans. Moreover, in GALT species, these cells must produce a complete B cell repertoire whereas, in humans, their task is limited to the high-affinity antibody response to highly pathogenic encapsulated bacteria with cross-reactive glycan structures. Nevertheless, these two processes, repertoire formation in GALT species or T-independent responses in humans, appear as two faces of the same coin, since, in both cases, a B cell repertoire diversified by mitotic or antigenic stimulation in gut-associated lymphoid tissues is used at the systemic level in a different, unrelated antigenic context.
